# Artificial Intelligence in Low-Dose Computed Tomography Lung Cancer Screening: Clinical Integration, Validation, and Translational Challenges

**DOI:** 10.7759/cureus.107050

**Published:** 2026-04-14

**Authors:** Valeria Vanessa Varela Betancourt, Archana Acharya, Nusrat Jahan, Udit Kapahi, Insharah Khalid, Syed M Shah, Hassan Ibrahim, Bushra Nawaz, Shazma Shayan, Maia Valls Palacios Reese, Sheeza Nadeem, Manju Rai

**Affiliations:** 1 General Medicine, Universidad Nacional de Colombia, Bogota, COL; 2 General Surgery, Sri Ramachandra Bhanja Medical College, Cuttack, IND; 3 Internal Medicine, Kent and Medway Mental Health NHS Trust, Kent, GBR; 4 Internal Medicine, Atal Bihari Vajpayee Institute of Medical Sciences and Dr. Ram Manohar Lohia Hospital, New Delhi, IND; 5 Internal Medicine, Nishtar Medical University, Multan, PAK; 6 Neuroscience, Kansas City University College of Osteopathic Medicine, Kansas, USA; 7 Internal Medicine, Darent Valley Hospital, Dartford, GBR; 8 Medicine and Surgery, Islamic International Medical College, Rawalpindi, PAK; 9 Internal Medicine, Hull University Teaching Hospitals, Hull, GBR; 10 Internal Medicine, Ponce Health Sciences University, School of Medicine, Ponce, PRI; 11 Internal Medicine, Doncaster and Bassetlaw Hospital NHS Foundation Trust, Doncaster, GBR; 12 Medicine, Jinnah Medical and Dental College, Karachi, PAK; 13 Biotechnology, Shri Venkateshwara University, Uttar Pradesh, IND

**Keywords:** artificial intelligence, deep learning, low-dose computed tomography, pulmonary nodules, radiomics, risk prediction

## Abstract

Lung cancer remains the leading cause of cancer-related mortality worldwide, largely due to late-stage diagnosis. Low-dose computed tomography (LDCT) screening has demonstrated significant mortality reduction in high-risk populations; however, its widespread implementation is limited by high false-positive rates, inter-reader variability, and substantial workflow burden. Artificial intelligence (AI) has emerged as a promising adjunct to address these challenges by enhancing diagnostic consistency, efficiency, and risk stratification in LDCT-based screening. This narrative review aims to synthesize current evidence on AI methodologies, clinical applications, validation studies, and translational challenges in LDCT-based lung cancer screening. A structured literature search was conducted across PubMed, Scopus, Embase, and the Cochrane Library for studies published between January 2010 and September 2025, using relevant keywords related to AI, radiomics, and lung cancer screening. Studies were selected based on their focus on AI applications in LDCT, including detection, characterization, risk prediction, and workflow optimization. Recent advances in deep learning and radiomics have enabled automated detection, segmentation, and characterization of pulmonary nodules with performance comparable to expert radiologists. Hybrid AI models that integrate imaging-derived features with clinical and demographic data further improve individualized risk prediction and support tailored screening strategies. AI-supported workflows have demonstrated improved efficiency by reducing interpretation time while maintaining diagnostic accuracy. Despite these advances, translation into routine clinical practice remains inconsistent due to limitations in external validation, generalizability, interpretability, and workflow integration. Radiologists' trust and human-AI interaction further influence real-world adoption. This review highlights the need to shift focus from algorithmic performance to clinical integration and human-AI collaboration to ensure meaningful improvements in lung cancer screening outcomes.

## Introduction and background

Lung cancer remains the leading cause of cancer-related mortality worldwide, accounting for an estimated 1.8 million deaths in 2020 [1]. Despite therapeutic advances, the overall 5-year survival rate remains dismal at approximately 17%, largely due to late-stage diagnoses [2]. In contrast, patients diagnosed with stage I disease have a 5-year survival rate approaching 70%, underscoring the critical importance of early detection in improving long-term outcomes.

Low-dose computed tomography (LDCT), a specialized imaging technique that uses reduced radiation doses to detect early lung abnormalities, has emerged as a pivotal tool for lung cancer screening in high-risk populations. Landmark studies demonstrated significant reductions in lung cancer-specific mortality, leading to the incorporation of LDCT into international screening recommendations [3-5]. By facilitating earlier detection of potentially curable disease, LDCT has fundamentally reshaped lung cancer screening paradigms.

However, widespread LDCT implementation has revealed significant operational and diagnostic challenges, including high false-positive rates, reported to be approximately 20-25% per screening round in large trials such as the National Lung Screening Trial (NLST), along with inter-reader variability and substantial workflow burden [6-7]. These limitations constrain scalability, increase downstream investigations, and contribute to patient anxiety, underscoring the need for adjunctive strategies that enhance diagnostic precision and efficiency.

Across clinical medicine, artificial intelligence (AI) has demonstrated the potential to improve diagnostic ‎accuracy, support therapeutic decision-making, enhance operational efficiency, and strengthen ‎patient safety across diverse applications [8]. AI, particularly deep learning-based algorithms that can automatically learn patterns from medical images, offers promising solutions to many of the operational and diagnostic challenges inherent to LDCT screening. Advanced AI systems can autonomously detect, segment, and classify pulmonary nodules on CT images with high sensitivity and specificity, in some cases matching or exceeding expert radiologist performance [8-9]. When deployed as adjunct “second readers,” AI systems have the potential to standardize interpretations, improve workflow efficiency, and reduce cognitive burden on radiologists [10]. Collectively, these advances position AI as a powerful enabler of more precise, scalable, and data-driven lung cancer screening.

Despite rapid technical progress, the clinical translation of AI for LDCT-based lung cancer screening remains inconsistent. High algorithmic accuracy has not uniformly translated into improved screening outcomes, largely due to challenges in external validation, workflow integration, interpretability, and human-AI interaction. In particular, radiologists' trust, the real-world utility of interpretability methods, and the impact of AI on diagnostic decision-making remain underexplored. This narrative review, therefore, moves beyond performance metrics to critically examine the translational barriers limiting real-world adoption of AI in LDCT screening, with a focus on clinical integration, validation realism, and human-machine collaboration.

## Review

Methodology

Search Strategy and Data Sources

This narrative review was conducted to synthesize current evidence on the integration of AI in low-dose computed tomography (LDCT) for lung cancer screening. A comprehensive literature search was performed across PubMed, Scopus, Embase, and the Cochrane Library for publications from January 2010 to September 2025. The search combined MeSH terms and keywords, including “Artificial Intelligence,” “Machine Learning,” “Deep Learning,” “Radiomics,” “Low-Dose Computed Tomography,” “Lung Neoplasms,” “Pulmonary Nodules,” “Screening,” “Risk Prediction,” and “Prognosis.” Boolean operators (“AND,” “OR”) were applied to refine retrieval.

Study Selection and Eligibility Criteria

**Figure 1:**
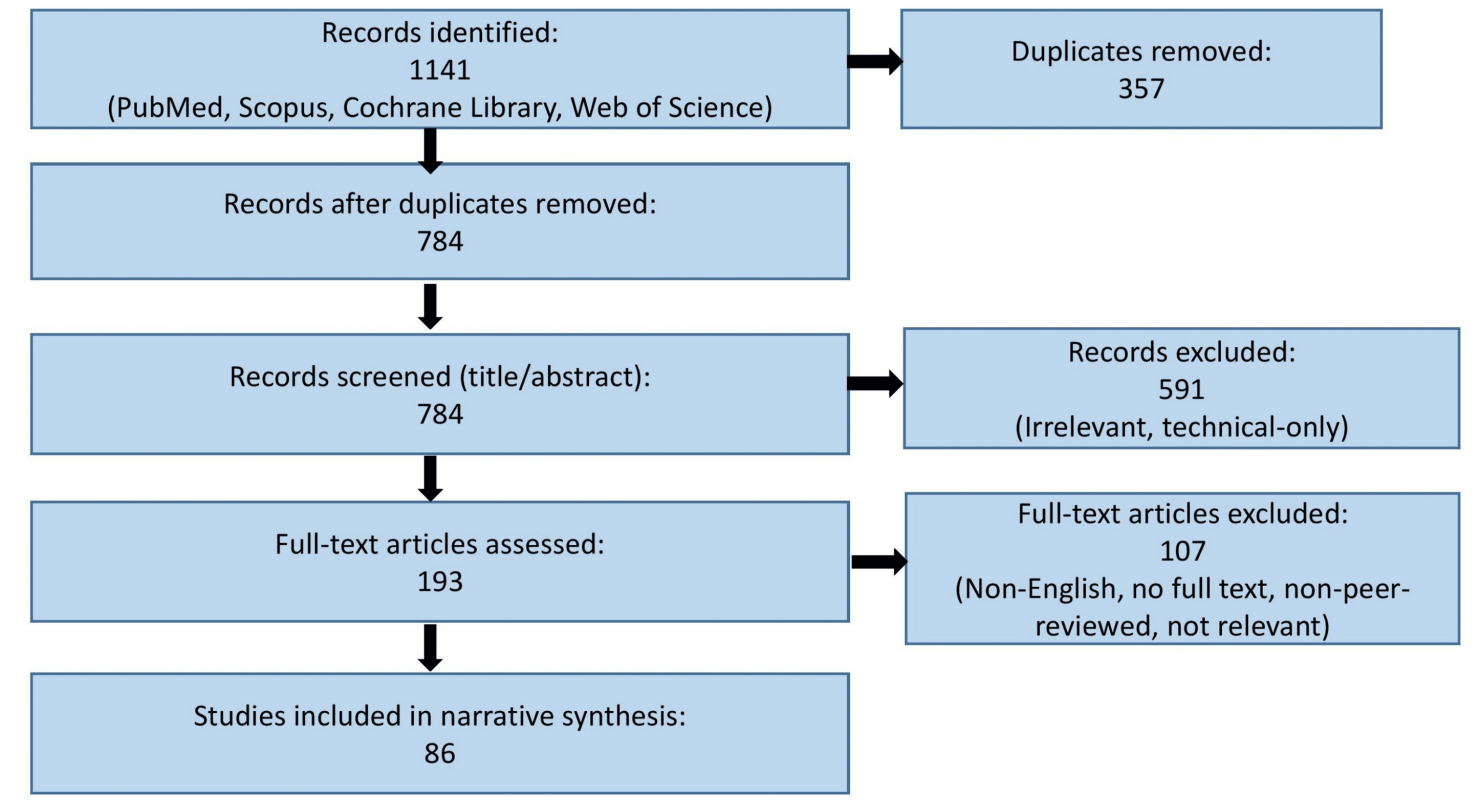
Flow diagram summarizing the study selection process for the included literature in this comprehensive review Figure created using PowerPoint (Microsoft Corporation, Redmond, USA).

Studies were included if they examined AI or radiomics applications in LDCT-based lung cancer screening, focusing on detection, characterization, risk prediction, or workflow optimization. Exclusion criteria encompassed non-human studies, abstracts, editorials, and papers unrelated to LDCT or AI. Figure 1 presents a flow diagram illustrating the literature identification, screening, eligibility assessment, and inclusion process for studies considered in this review.

Data Synthesis Approach and Scope of Review

Elements of the Preferred Reporting Items for Systematic Reviews and Meta-Analysis (PRISMA) framework were applied to enhance transparency in literature identification and selection. However, given the narrative design, a formal risk-of-bias assessment was not performed; however, emphasis was placed on including clinically relevant and methodologically robust studies.

Current landscape of lung cancer screening

Lung cancer screening using LDCT is now an established strategy for detecting early-stage disease in high-risk populations. Its clinical adoption is guided by recommendations from major professional organizations, including the U.S. Preventive Services Task Force (USPSTF) and the National Comprehensive Cancer Network (NCCN), informed by evidence from large randomized controlled trials demonstrating meaningful mortality reduction.

Screening Guidelines

The USPSTF recommends annual LDCT screening for adults aged 50-80 years with a smoking history of at least 20 pack-years, including current smokers and those who have quit within the past 15 years. Screening is discontinued once an individual has abstained from smoking for 15 years or develops a comorbidity that precludes curative treatment [5]. The NCCN issues similar age- and exposure-based recommendations but adopts a more individualized approach by incorporating additional risk modifiers such as chronic obstructive pulmonary disease, occupational carcinogen exposure, and prior thoracic malignancy [11]. Although factors such as family history and passive smoke exposure contribute to overall risk, they are not currently used as independent criteria for screening eligibility.

Evidence From Key Trials

The clinical efficacy of LDCT screening is supported by several landmark trials. The NLST demonstrated a 20% reduction in lung cancer-specific mortality compared with chest radiography, establishing LDCT as a superior screening modality [12,13]. The European Nederlands-Leuvens Longkanker Screenings Onderzoek (NELSON) trial corroborated these findings, reporting mortality reductions of 24% in men and 33% in women screened with LDCT versus no screening [14]. The UK Lung Cancer Screening (UKLS) trial further validated LDCT’s effectiveness in a European population and provided important insights into the feasibility of population-based screening implementation [12]. Collectively, these trials form the evidentiary foundation for current international screening recommendations [13].

Limitations of LDCT Screening

Despite its proven mortality benefit, several limitations continue to restrict the widespread and equitable implementation of LDCT screening. High false-positive rates lead to unnecessary diagnostic procedures, psychological distress, and increased healthcare costs [4,13]. Repeated annual imaging raises concerns regarding cumulative radiation exposure, albeit at relatively low individual doses [14]. Incidental nonmalignant findings are common and often require additional follow-up, further increasing clinical workload. The operational burden on radiology services, particularly in low- and middle-income countries with constrained infrastructure and workforce capacity, represents an additional barrier to scale [15]. Together, these limitations underscore the need for innovations that can enhance diagnostic precision, reduce inefficiencies, and support sustainable screening workflows, providing a clear rationale for the growing interest in AI-driven solutions.

Artificial intelligence methodologies in LDCT for lung cancer

Deep Learning Approaches for Nodule Detection and Segmentation

The integration of AI into LDCT screening has reshaped pulmonary nodule detection and characterization by improving diagnostic consistency, efficiency, and quantitative assessment. Conventional LDCT interpretation is constrained by inter-reader variability and increasing workload, whereas AI systems can learn complex imaging patterns and support standardized analysis across large screening cohorts. Two complementary methodological paradigms - deep learning and radiomics (which involves the extraction of quantitative imaging features such as shape, texture, and intensity from medical images) - form the foundation of most contemporary AI applications in LDCT-based lung cancer screening.

Deep Learning Approaches for Nodule Detection: Deep learning models, predominantly based on convolutional neural networks (CNNs), have demonstrated strong performance in pulmonary nodule detection, segmentation, and classification. CNN-based architectures process imaging data through hierarchical feature extraction, enabling the recognition of subtle morphological patterns associated with malignancy that may be challenging to identify consistently in manual interpretation.

Early approaches relied on two-dimensional slices or patch-based three-dimensional inputs, whereas more recent models incorporate volumetric analysis and multi-scale contextual learning. Segmentation-optimized architectures such as U-Net variants and residual networks (ResNets) are commonly used for nodule delineation, while region proposal-based frameworks facilitate candidate localization [16-18]. Importantly, most published systems employ modular pipelines, in which detection, segmentation, and classification are trained and optimized separately, rather than fully end-to-end models.

Representative studies illustrate the potential of these approaches. Lee et al. developed a deep learning system integrating radiologic and clinical features to predict the invasiveness of ground-glass nodules, demonstrating high concordance with histopathologic outcomes [19]. Other architectures, including DenseVNet and attention-based networks such as Lung_PAYNet, have improved segmentation accuracy and robustness across nodule types [20,21]. Three-dimensional CNN frameworks further enhance volumetric analysis and small-nodule detection, achieving radiologist-comparable sensitivity in NLST-derived cohorts [22,23]. Despite these advances, performance remains sensitive to training data composition and validation strategy, limiting immediate generalizability.

Radiomics for Nodule Characterization: Radiomics complements deep learning by transforming medical images into high-dimensional quantitative feature sets that describe lesion shape, texture, and intensity heterogeneity. A typical radiomics workflow includes image acquisition, region-of-interest segmentation, handcrafted feature extraction, feature selection using statistical or machine learning techniques, such as least absolute shrinkage and selection operator (LASSO), and predictive model construction [24,25].

Radiomics-based models have demonstrated utility in predicting nodule invasiveness, growth kinetics, and histologic subtype. For example, Zhang et al. developed a CT-based radiomics model capable of distinguishing invasive adenocarcinoma among subcentimeter subsolid nodules, while Liu et al. incorporated radiomic and clinical variables into a malignancy prediction nomogram [26,27]. Features such as spiculation, lobulation, and perinodular texture gradients have shown consistent associations with malignancy risk [28,29]. However, radiomics approaches remain sensitive to imaging protocol variability and segmentation reproducibility, posing challenges for large-scale screening deployment.

Integration and Complementarity

**Figure 2:**
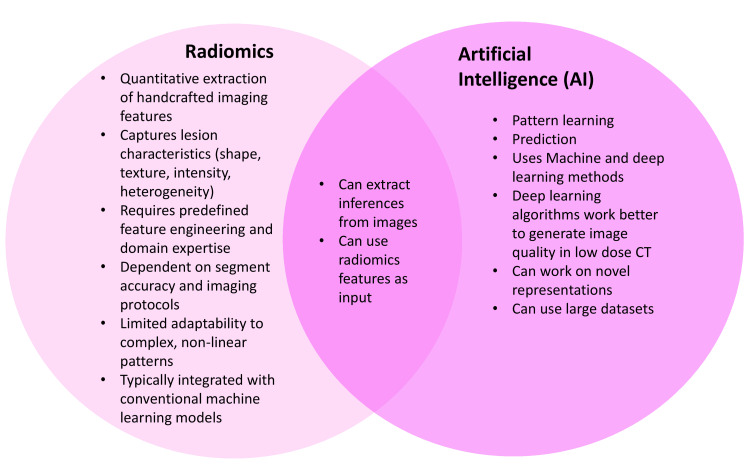
Comparison between radiomics and artificial intelligence (AI) in medical imaging Radiomics focuses on the extraction of handcrafted quantitative imaging features that describe lesion shape, texture, and intensity, often requiring machine learning for analysis. Artificial intelligence, particularly deep learning, autonomously learns patterns from large imaging datasets for prediction and classification. The intersection highlights their complementarity: AI can utilize radiomic features as input to enhance image-based inference and predictive modeling. Figure has been created in Biorender.com without any AI help (BioRender, Toronto, Canada).

Deep learning and radiomics are increasingly used in combination rather than isolation. While CNNs enable automated feature learning directly from imaging data, radiomics provides explicit, handcrafted descriptors that may enhance transparency and hypothesis-driven interpretation. Hybrid deep learning-radiomics pipelines aim to leverage the strengths of both approaches, enabling automated feature extraction while retaining clinically interpretable metrics. This complementary strategy supports more comprehensive nodule assessment, although it does not eliminate the need for rigorous external validation. A conceptual comparison of radiomics and AI methodologies is illustrated in Figure 2. 

Hybrid Models: Concept and Advantages

Beyond image-only analysis, hybrid AI models integrate imaging-derived features with clinical and epidemiological risk factors to generate individualized risk estimates. By combining CNN-based outputs (e.g., nodule probability, size, and volumetrics) with variables such as age, smoking history, and comorbidities, these models consistently outperform image-only or clinical-only approaches, with reported discrimination often approaching an area under the curve (AUC) of 0.9 in externally validated cohorts [30].

For instance, a co-learning framework trained on NLST imaging data and validated in the Vanderbilt Lung Screening Program cohort demonstrated superior risk discrimination compared with unimodal models [30]. Similarly, the NLST-University of Illinois Health (UIH) hybrid model improved six-year lung cancer risk prediction by integrating ResNet-derived imaging scores with clinical predictors [31]. Established clinical risk models, including the PanCan and PLCOm2012 frameworks, further illustrate how quantitative imaging features can refine malignancy probability estimates and guide management decisions such as surveillance versus diagnostic intervention [32].

Datasets for Training and Validation

Progress in LDCT-based AI has been enabled by publicly available datasets, yet methodological performance remains tightly coupled to dataset characteristics. Benchmarks such as LIDC-IDRI facilitate algorithm development through rich annotations but lack robust longitudinal outcomes, whereas screening trials such as NLST provide clinically meaningful endpoints at the cost of imaging heterogeneity [3,33]. Curated datasets like LUNA16 support reproducible benchmarking but offer limited clinical metadata [34]. Consequently, model performance reported under controlled conditions may not reflect real-world screening populations.

Across methodologies, dataset diversity, realistic disease prevalence, and external validation across independent cohorts are essential to mitigate spectrum bias and domain shift. Without these safeguards, AI systems risk overfitting to site-specific artifacts and underperforming when deployed in routine LDCT screening workflows.

Applications of AI in LDCT for lung cancer screening

Nodule Detection and Segmentation

Nodule detection and segmentation constitute the foundational applications of AI in LDCT screening, directly influencing diagnostic accuracy, workflow efficiency, and longitudinal monitoring. In clinical practice, missed nodules and inconsistent size measurements remain major contributors to delayed diagnosis and variability in follow-up. AI-assisted systems address these challenges by providing automated, reproducible identification and delineation of pulmonary nodules across entire lung volumes.

Convolutional neural networks-based architectures, including segmentation-optimized designs such as U-Net and nnU-Net, as well as classification-oriented residual architectures (e.g., ResNet variants) and volumetric 3D CNN implementations, have demonstrated robust performance in automating these processes [35-37]. These algorithms leverage hierarchical feature extraction to capture subtle textural and morphological patterns, enabling the identification of small, low-contrast nodules often missed in manual readings [36]. Automated segmentation additionally facilitates reproducible volumetric analysis, essential for assessing nodule growth over serial scans and differentiating between true progression and measurement variability.

**Figure 3:**
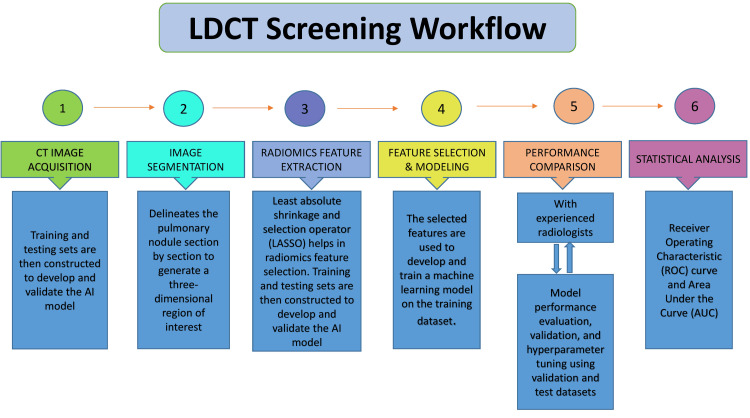
LDCT Screening Workflow Schematic representation of the workflow used in low-dose computed tomography (LDCT)-based lung cancer screening. The process begins with CT image acquisition from screening cohorts or existing databases, followed by image segmentation to delineate pulmonary nodules. Radiomics feature extraction quantifies image characteristics, which are refined through feature selection methods such as LASSO to identify predictive biomarkers. These selected features are then used to develop and train artificial intelligence models, whose performance is validated and compared with expert radiologists using receiver operating characteristic (ROC) curves and area under the curve (AUC) analysis. Figure created in Biorender.com without any AI help (Biorender, Toronto, Canada). LIDC-IDRI: Lung Image Database Consortium and Image Database Resource Initiative

Across multiple benchmark studies, AI-based detection systems have achieved sensitivities between 85% and 98%, with false-negative rates lower than traditional computer-aided detection (CADe) methods [38]. Such efficiency not only reduces radiologist workload but also minimizes diagnostic delays and enhances consistency across readers and institutions. The overall workflow of AI-assisted LDCT screening, from image acquisition to model validation, is depicted in Figure 3.

Nodule Characterization and Malignancy Assessment

Beyond detection, AI-based characterization tools aim to distinguish benign from malignant nodules and to identify features associated with aggressive behavior. Although contemporary literature often refers to “AI pipelines” for pulmonary nodule characterization, the majority of published approaches employ modular workflows rather than fully end-to-end systems. In many studies, nodule detection and segmentation are performed using semi-automated or rule-based tools, followed by separate radiomic feature extraction or machine-learning classifiers [26,36,38].

Artificial intelligence-enabled characterization has demonstrated utility in estimating malignancy probability, assessing growth kinetics, and differentiating invasive from indolent lesions. Integration of clinical variables such as age and smoking history with imaging-derived features consistently improves predictive performance compared with image-only models [39-40]. Radiogenomic investigations have further shown that certain radiomic patterns correlate with molecular alterations, suggesting a potential role for AI in guiding personalized management strategies [40].

However, characterization performance remains sensitive to image acquisition variability and segmentation quality, underscoring the need for cautious interpretation and external validation. In routine clinical practice, AI-derived malignancy estimates are best used to augment radiologist judgment rather than as standalone decision-makers.

Artificial intelligence-driven risk prediction models

Artificial intelligence-based risk prediction represents a major advancement in lung cancer screening by enabling individualized estimation of disease probability. Unlike diagnostic algorithms focused on lesion-level classification, risk prediction models integrate imaging biomarkers with demographic, behavioral, and clinical variables to forecast a patient’s future likelihood of developing lung cancer. This approach supports adaptive screening intervals, targeted surveillance, and precision prevention strategies [30].

Hybrid models combining LDCT-derived radiomic signatures with epidemiological data have consistently outperformed traditional regression-based risk scores such as PLCOm2012. A recent meta-analysis of externally validated models reported a pooled AUC of 0.82 for AI-based frameworks, compared with 0.73 for conventional risk equations [41]. Among imaging-clinical hybrids, the Sybil model demonstrated that a single LDCT scan could predict six-year lung cancer risk without requiring longitudinal data, achieving AUCs exceeding 0.86 on external validation [42]. A recent study evaluating machine learning-based risk prediction models demonstrated that stacking ensemble and LightGBM approaches outperformed traditional logistic regression, achieving AUCs of up to 0.887, thereby highlighting the potential of advanced machine learning techniques to enhance lung cancer risk prediction using epidemiological data [41]. Complementary approaches using non-imaging data, such as EHR-based machine learning models, also highlight the potential of AI to dynamically integrate multimodal data for personalized risk assessment [43,44].

Workflow Optimization and Clinical Integration

Within radiology workflows, AI serves as a concurrent reader by triaging LDCT scans, flagging suspicious nodules, and generating structured reports with standardized terminology. This automation reduces interpretation time, mitigates inter-reader variability, and supports consistent documentation [45]. In the UK Lung Screening (UKLS) trial, an AI-assisted platform demonstrated diagnostic performance equivalent to expert radiologists, achieving a negative predictive value of 99.8% while reducing reading workload by 67-79% [46].

 Models incorporating synthetic data augmentation and machine learning classifiers, such as CTGAN-based frameworks combined with random forest algorithms, have demonstrated improved classification performance and robustness, particularly in handling class imbalance and enhancing prediction accuracy in lung cancer detection[47]. Together, AI-driven risk prediction and workflow optimization form a complementary continuum: the former informs who should be screened and when, while the latter improves how screening is executed. A summary of representative studies evaluating AI applications in LDCT-based lung cancer screening is presented in Table 1.

**Table 1:** Summary of Major AI Applications in LDCT-Based Lung Cancer Screening AI: artificial intelligence; CNN: convolutional neural network; CT: computed tomography; LDCT: low-dose computed tomography; NLST: National Lung Screening Trial; LIDC-IDRI: Lung Image Database Consortium and Image Database Resource Initiative; MGH: Massachusetts General Hospital; UKLS: United Kingdom Lung Cancer Screening Trial; AUC: area under the receiver operating characteristic curve.

AI Application	Representative Model/Study	Key Dataset Used	Primary Function	Performance (AUC/ Sensitivity/Specificity)	Clinical Implication
Nodule Detection	Ardila et al., 2019 [8]	NLST	End-to-end 3D CNN for early cancer detection	AUC 0.94	Radiologist-level malignancy prediction
Nodule Segmentation	Astaraki et al., 2021 [21]	LIDC-IDRI	Pyramidal attention–based segmentation (Lung_PAYNet)	Dice = 0.89	Improved boundary delineation and reproducibility
Nodule Characterization	Zhang et al., 2023 [26]	Multi-institutional cohort	CT-based radiomics for histologic invasiveness	AUC 0.87	Differentiation of invasive adenocarcinoma
Risk Prediction	Ardila et al., 2019 [8]	NLST, MGH, Chang Gung	Deep learning for 6-year risk estimation	AUC 0.86–0.91	Predicts future cancer risk from a single LDCT
Workflow Optimization	Lancaster et al., 2025 [46]	UKLS	AI as a concurrent reader	Workload ↓ 67–79%	Efficient triage; equivalent accuracy to experts

Clinical Impact and Remaining Gaps

Collectively, AI applications in LDCT screening enhance detection consistency, support refined risk stratification, and improve operational efficiency. However, their real-world clinical impact remains constrained by variability in validation quality, limited prospective evidence, and an incomplete understanding of how AI influences radiologist decision-making.

Current evidence suggests that AI delivers its greatest value when deployed as a decision-support system that augments human expertise rather than replaces it [10,46]. Addressing remaining gaps, particularly in external validation, interpretability, and human-AI interaction, will be essential to translating algorithmic performance gains into meaningful improvements in lung cancer screening outcomes.

Clinical evidence and validation of AI

Evidence from Validation Studies and Clinical Trials

Following the establishment of LDCT as an effective modality for reducing lung cancer mortality, subsequent research has focused on validating AI systems within screening workflows to assess diagnostic performance, operational impact, and clinical feasibility [8-10]. Across retrospective and prospective cohorts, AI-assisted LDCT screening has consistently demonstrated improved sensitivity for cancer detection, particularly for early-stage disease [46,48,49].

In a large-scale evaluation, Harlianto and de Jong reported that AI integration identified an estimated 150-750 additional cancers per million screened individuals compared with conventional interpretation [48]. While these gains highlight AI’s potential to enhance case-finding efficiency, they were accompanied by modest reductions in specificity, resulting in increased downstream surveillance and follow-up imaging. Similar findings were observed in a 2024 multicenter study combining CT-based radiomics with clinical variables, which demonstrated improved early cancer detection but at the cost of higher false-positive rates [49]. Collectively, these studies illustrate a recurring pattern: AI enhances sensitivity but requires careful triage strategies to prevent unnecessary diagnostic escalation.

Importantly, most validation studies remain retrospective and are conducted in enriched or controlled datasets, limiting their ability to fully capture real-world screening dynamics such as incidental findings, patient anxiety, and longitudinal management decisions.

Comparative Performance: AI vs Radiologists

Direct comparisons between AI systems and expert radiologists provide further insight into clinical applicability. In the UK Lung Cancer Screening Trial (UKLS), AI-assisted interpretation demonstrated diagnostic concordance with a European expert panel and produced fewer interpretive discrepancies than individual readers [46]. When deployed as a concurrent reader, AI reduced radiologist workload by up to 86% without compromising diagnostic accuracy [50].

Model-specific evaluations reinforce these findings. The Optimized Early Warning for Lung Cancer Risk (OWL) model, validated across the UK Biobank, Prostate, Lung, Colorectal, and Ovarian (PLCO) Cancer Screening Trial, and NLST cohorts, outperformed traditional risk models such as PLCOm2014 and LLPv3 in selected populations, indicating reasonable cross-cohort generalizability [51]. Similarly, the Sybil model demonstrated consistent performance across NLST, Massachusetts General Hospital, and Chang Gung cohorts, predicting future lung cancer risk from a single LDCT scan in real time within standard radiology workflows [52].

Despite these encouraging results, AI-radiologist comparisons consistently show that AI performs best as a decision-support tool rather than a standalone reader. Discrepancies tend to arise in borderline or indeterminate nodules, reinforcing the necessity of radiologist oversight and contextual clinical interpretation.

Cost-Effectiveness and Clinical Scalability

Economic evaluations provide an additional dimension to validation by assessing whether AI-assisted screening can be sustainably scaled. Cost-effectiveness analyses from the United States and Australia suggest that AI-supported LDCT screening could reduce overall screening-related costs by approximately 30-40%, primarily through reduced reading time, automated segmentation, and more efficient case triage [53-54]. The UKLS experience similarly estimated that first-reader AI implementation could decrease CT interpretation workload by 67-79% while maintaining a high negative predictive value of 99.8% [46].

These findings indicate that AI has the potential to improve both clinical efficiency and economic viability, particularly in high-volume screening programs. However, most cost-effectiveness models assume stable algorithm performance and seamless workflow integration, assumptions that may not hold in heterogeneous real-world settings.

Validation Gaps and Real-World Readiness

Despite growing evidence supporting AI-assisted LDCT screening, important validation gaps remain. Most systems have not undergone prospective evaluation within routine clinical workflows, and few studies assess downstream outcomes such as changes in radiologist behavior, referral patterns, or patient-level outcomes. Moreover, external validation across diverse populations, scanners, and healthcare systems remains inconsistent.

Current evidence suggests that AI systems are technically mature but clinically fragile when deployed outside development environments. Without standardized validation frameworks, continuous performance monitoring, and clear integration pathways, algorithmic gains in sensitivity may not translate into improved screening outcomes. Addressing these gaps will require prospective trials, harmonized benchmarking, and explicit evaluation of human-AI interaction within real-world screening programs.

Integration of AI into clinical practice

Low-dose computed tomography remains the cornerstone of lung cancer screening for high-risk individuals [3,55-56]. Although advances in AI-assisted LDCT interpretation have improved diagnostic consistency, inter-reader variability remains a challenge, particularly in pulmonary nodule detection and characterization. AI-based systems have demonstrated high agreement with expert radiologists, supporting their role in reducing variability[57]. AI has therefore emerged as an adjunctive tool designed to enhance detection efficiency, reduce observer variability, and support standardized interpretation within existing clinical workflows.

Artificial intelligence systems can be integrated at multiple points along the LDCT screening pathway. As a prescreening reader, AI algorithms automatically evaluate LDCT scans to identify examinations with a very low likelihood of malignancy, allowing radiologists to prioritize higher-risk cases [58]. As a concurrent reader, AI operates alongside the radiologist, highlighting candidate nodules, suggesting Lung-RADS categories, and generating structured quantitative outputs to support reporting [59,60]. Importantly, these systems are designed to integrate seamlessly with Picture Archiving and Communication Systems (PACS) and Radiology Information Systems (RIS), minimizing disruption to established clinical operations.

Impact on Workflow Efficiency and Consistency

Clinical studies have demonstrated that AI-assisted LDCT interpretation can substantially reduce reading time while maintaining diagnostic accuracy. Reported workload reductions range from 60% to 80% in high-volume screening environments, largely due to automated triage, standardized measurements, and reduced manual segmentation [57,59,60]. In the context of national or population-based screening programs, such efficiency gains are critical for managing increasing imaging volumes without proportional expansion of the radiology workforce.

Within structured screening frameworks such as the NELSON trial, AI-assisted workflows incorporating semi-automated segmentation and quantitative analysis improved the reproducibility of established risk stratification tools, including the Mayo Clinic, PanCan, and UKLS models [61]. These findings suggest that AI integration can enhance consistency across readers and institutions, particularly when applied to longitudinal nodule assessment.

Practical Constraints and Implementation Considerations

Despite demonstrated efficiency gains, successful clinical integration of AI depends on careful system design and governance. Over-alerting, poorly calibrated thresholds, or non-intuitive interfaces may contribute to alert fatigue or inappropriate reliance on algorithmic outputs. Furthermore, AI performance may degrade when deployed across institutions with differing scanners, acquisition protocols, or patient demographics, underscoring the need for site-specific calibration and ongoing performance monitoring.

In low- and middle-income countries, infrastructural limitations-including limited access to advanced CT scanners, computing resources, and trained radiologists-pose additional challenges to AI deployment [62-63]. While mobile LDCT units and lightweight AI platforms have shown feasibility in pilot studies, long-term scalability and cost-effectiveness remain to be established [62]. These constraints highlight that AI integration is not solely a technical exercise, but a health-system-level intervention requiring alignment with local resources and priorities.

Positioning AI as Clinical Decision Support

Across implementation scenarios, AI is most effective when positioned as a decision-support system, augmenting radiologist expertise rather than replacing it. Radiologists retain responsibility for synthesizing AI-generated outputs with clinical context, patient history, and multidisciplinary input. This human-centered deployment model minimizes the risk of automation bias while preserving the efficiency gains offered by AI.

Crucially, successful integration depends not only on algorithmic performance but also on clinician acceptance, transparency of outputs, and clarity of accountability. These factors directly influence how AI recommendations are incorporated into diagnostic decision-making, forming the basis for trust-building and sustained adoption, issues explored in the following section on "Human-AI Collaboration and Trust Building."

Human-AI Collaboration and Trust Building

Among all barriers to the clinical adoption of AI in LDCT-based lung cancer screening, radiologist interaction and trust represent the most decisive, and least algorithmically solvable, challenges. While AI systems increasingly demonstrate high diagnostic accuracy, their real-world effectiveness depends on how clinicians interpret, contextualize, and act upon algorithmic outputs within routine practice.

Emerging evidence indicates that radiologists' trust in AI systems directly influences diagnostic behavior and downstream clinical decision-making. Studies examining human-AI interaction have shown that low trust in algorithmic recommendations is associated with increased rates of discordant interpretations, whereas calibrated trust improves diagnostic agreement and workflow efficiency. Rainey et al. (2024) demonstrated that the presentation format of AI outputs-including saliency activation maps and binary classification results-significantly influenced radiologist confidence, inter-reader consensus, and willingness to incorporate AI recommendations into reporting decisions [64]. Importantly, excessive or poorly contextualized explanations were shown to paradoxically reduce trust, highlighting that interpretability must align with clinical reasoning rather than purely technical transparency.

Despite increasing automation, radiologists remain the final arbiters in AI-augmented LDCT screening. Clinicians integrate AI-generated outputs with patient-specific context, including smoking history, comorbidities, prior imaging, and multidisciplinary input, to arrive at management decisions [58]. In this setting, AI functions most effectively as a decision-support tool that enhances perceptual accuracy and consistency while preserving human oversight.

Standardization enabled by AI may also foster trust in collaborative environments such as double reading and multidisciplinary tumor boards. By reducing inter-observer variability and providing reproducible quantitative metrics, AI-supported workflows can improve consensus across readers and institutions [60]. However, inappropriate calibration of trust-either over-reliance on AI or systematic dismissal of its outputs-poses clinical risks, emphasizing the need for structured training and governance frameworks that define the role and limitations of AI assistance.

Crucially, algorithm transparency alone does not guarantee clinician trust. Although saliency activation maps and similar explainability tools are frequently proposed as mechanisms to enhance transparency, their clinical utility remains limited. These visualizations often require subjective interpretation, demonstrate inter-reader variability, and may not reliably convey the causal reasoning underlying model predictions. As such, interpretability methods should be regarded as adjunctive aids rather than definitive explanations of algorithmic behavior. Trust in clinical AI is therefore more strongly shaped by consistent performance across populations, clear failure modes, and demonstrated impact on diagnostic outcomes than by visual explainability alone.

Ultimately, successful human-AI collaboration depends less on further improvements in algorithmic accuracy and more on aligning AI systems with radiologists’ cognitive workflows, decision thresholds, and accountability structures. Embedding AI within transparent governance models and continuously evaluating its influence on clinical behavior will be essential to achieving sustained adoption and meaningful improvements in LDCT-based lung cancer screening.

Challenges and limitations in AI-enhanced LDCT screening

Despite substantial advances in AI-assisted LDCT screening, multiple unresolved challenges continue to limit consistent clinical translation. These barriers span data quality, generalizability, interpretability, and health-system readiness, underscoring that algorithmic performance alone is insufficient for real-world adoption.

Data Quality, Bias, and Representativeness

A persistent limitation of AI models for LDCT screening lies in the quality and representativeness of training data. Many systems are developed using retrospective or single-center datasets enriched with malignant nodules, which do not reflect the low disease prevalence encountered in population-based screening [65]. This enrichment inflates apparent performance and contributes to spectrum bias, limiting applicability to routine clinical settings.

Incomplete histopathologic confirmation further complicates model evaluation, as many studies rely on imaging follow-up rather than tissue diagnosis as the reference standard [66]. Variability in CT acquisition parameters, reconstruction kernels, and annotation practices across institutions also undermines reproducibility and contributes to performance degradation when models are deployed outside their development environment [67]. Collectively, these factors restrict confidence in reported metrics and impede regulatory and clinical acceptance.

Generalizability Across Populations and Imaging Environments

Transferability across diverse populations and healthcare systems remains a major unresolved challenge. Most AI models have been trained and validated predominantly in high-income countries using cohorts composed largely of White, heavy-smoking individuals, with limited representation of women, non-smokers, and ethnically diverse populations [65]. Differences in scanner technology, imaging protocols, and definitions of malignancy risk further impair model robustness when applied across institutions or regions [68].

These limitations are particularly pronounced in low- and middle-income countries, where lung cancer incidence is rising, but validation data are scarce [62,63,69]. AI frameworks often assume access to advanced imaging infrastructure, computational resources, and trained radiologists - conditions that may not be met in resource-constrained settings. Without deliberate inclusion of underrepresented populations and region - specific calibration, AI deployment risks reinforcing existing disparities in lung cancer detection and outcomes [62,63].

Algorithm Transparency and Interpretability

The opacity of deep learning models remains a central barrier to clinician trust and regulatory approval. Although interpretability techniques such as saliency activation maps (e.g., Grad-CAM) are frequently proposed to enhance transparency, their clinical utility is limited [70,71]. These methods require subjective interpretation, demonstrate substantial inter-reader variability, and do not reliably reflect causal decision logic. As a result, saliency-based explanations may provide visual reassurance without meaningfully improving diagnostic confidence or decision quality.

This disconnect highlights a critical translational gap between technical explainability and clinically actionable understanding. Interpretability approaches that fail to influence clinician behavior or patient outcomes offer limited value in screening contexts, where decisions carry downstream consequences such as invasive testing or prolonged surveillance. Future validation efforts must therefore assess interpretability in terms of its impact on radiologist decision-making rather than visual plausibility alone.

Cost, Infrastructure, and Adoption Barriers

Economic and infrastructural constraints further limit large-scale adoption of AI-assisted LDCT screening. Implementation requires sustained investment in imaging hardware, computational infrastructure, software maintenance, and workforce training [62]. In LMICs, where screening programs themselves are often underfunded, the introduction of AI platforms may compete with essential healthcare priorities rather than complement them.

Although mobile LDCT units and lightweight AI systems have demonstrated feasibility in pilot studies, evidence supporting long-term cost-effectiveness, scalability, and integration into public health systems remains limited [62]. These considerations reinforce that AI deployment is not solely a technological challenge but a systems-level intervention requiring alignment with local clinical capacity, policy frameworks, and economic realities. The principal technical, ethical, and infrastructural barriers to large-scale clinical adoption of AI-assisted LDCT screening, along with potential strategies to overcome them, are summarized in Table 2.

**Table 2:** Key challenges and potential solutions for AI integration in lung cancer screening. AI: artificial intelligence; LDCT: low-dose computed tomography; CT: computed tomography; LMICs: low- and middle-income countries; NLST: National Lung Screening Trial; LIDC-IDRI: Lung Image Database Consortium and Image Database Resource Initiative; XAI: explainable artificial intelligence.

Challenge	Underlying Issue	Impact on Clinical Translation	Potential Solutions/Future Directions
Ethical and legal considerations	Data privacy and medico-legal uncertainty [62–63]	Hinders regulatory approval and adoption	Transparent governance, continuous post-market validation [63]
Data heterogeneity	Variations in CT acquisition, annotation, and reconstruction protocols [64-66]	Reduced reproducibility and external validity	Standardized imaging pipelines and harmonization tools [66]
Cost and infrastructure barriers	Limited computing and imaging capacity in LMICs [68–70]	Restricted scalability of AI-assisted LDCT	Lightweight cloud-based AI, mobile LDCT units [69]
Lack of interpretability	Deep learning models function as “black boxes” [65,67–69]	Low clinician trust, regulatory barriers	Explainable AI (XAI) frameworks, saliency mapping [71-72]
Limited generalizability	Models trained on Western, smoker-heavy datasets (NLST, LIDC-IDRI) [67-70]	Biased outputs in LMIC or non-smoker populations	Federated learning across global cohorts [70]

Future directions

AI-Driven Population Health Strategies

Future advances in AI-enabled LDCT screening will increasingly focus on population-level risk stratification and resource optimization. Predictive models that integrate demographic, clinical, behavioral, and environmental variables can identify individuals most likely to benefit from screening, enabling more targeted deployment of LDCT resources [26,72-74]. Such stratified approaches are particularly relevant in settings with limited imaging capacity, where indiscriminate screening is neither feasible nor cost-effective.

The development of AI-enabled registries linking electronic health records with regional imaging databases may further support dynamic population surveillance. By continuously learning from longitudinal data, these systems could enable adaptive screening intervals and personalized follow-up strategies, aligning lung cancer screening with emerging principles of precision public health [73-74].

Extending AI Beyond Detection: Prognosis and Treatment Planning

Beyond early detection, AI is increasingly being applied across the lung cancer care continuum. Radiomic-clinical ensemble models now quantify tumor heterogeneity and spatial phenotypes on CT and PET/CT imaging, correlating these features with histopathologic subtypes, recurrence risk, and treatment response [75-77]. In early-stage non-small cell lung cancer (NSCLC), AI-based prognostic tools have demonstrated the ability to predict recurrence and survival, informing decisions regarding adjuvant therapy intensity and surveillance schedules [75-78].

Artificial intelligence-assisted treatment planning is also gaining traction, particularly in radiotherapy. Automated contouring, dose optimization, and toxicity prediction models illustrate how AI can improve consistency and efficiency while reducing inter-operator variability [76]. These applications suggest a gradual shift from reactive imaging assessment toward anticipatory, data-informed oncology workflows.

Collaboration, Explainability, and Equity

As AI systems mature, the emphasis is shifting from standalone model performance to collaborative and equitable deployment. Federated and foundational learning frameworks enable multi-institutional model development without centralized data sharing, preserving patient privacy while enhancing generalizability [77,79,80]. Such approaches are particularly important for including institutions from low- and middle-income countries in global AI development efforts.

Explainable AI (XAI) remains an active area of research, driven by regulatory requirements and the need for clinician trust. However, future progress will depend on moving beyond visually intuitive explanations toward methods that demonstrably influence diagnostic behavior, decision confidence, and patient outcomes. Interpretability must therefore be evaluated as a clinical tool rather than a technical feature.

Equally critical is ensuring fairness and inclusivity in AI deployment. Addressing demographic bias, ensuring representation of diverse populations, and aligning model outputs with local clinical realities are essential to prevent AI from exacerbating existing healthcare disparities [79-80].

Multi-omics integration and Learning Health Systems

Artificial intelligence-driven lung cancer screening is poised to evolve beyond imaging-centric models through the integration of multi-omics data. Studies such as TRACERx demonstrate how genomic and microenvironmental heterogeneity underpins tumor evolution and clinical outcomes, providing a biological foundation for AI models that incorporate molecular signals alongside radiologic features [81]. Emerging multi-omics frameworks combining radiomics, genomics, and clinical data have already shown improved stratification of recurrence risk in early-stage NSCLC [82].

In the long term, these developments support the vision of a learning health system in which screening, diagnosis, treatment, and outcome data continuously inform model refinement. Federated learning infrastructures and international collaborations, such as the IASLC Early Lung Imaging Confederation, provide a scalable foundation for prospective validation and global benchmarking [83-86]. Together, these initiatives may enable AI systems that are not only accurate but also adaptive, equitable, and clinically meaningful across diverse healthcare environments.

## Conclusions

AI has emerged as a promising adjunct to low-dose computed tomography-based lung cancer screening, with the potential to enhance early detection, standardize interpretation, and improve workflow efficiency. Advances in deep learning, radiomics, and hybrid risk modeling have enabled more consistent nodule detection, refined malignancy risk stratification, and scalable screening operations, addressing several long-standing limitations of LDCT screening programs.

However, this review underscores that the principal barriers to successful AI adoption are no longer algorithmic performance or data availability, but clinical translation. Persistent challenges related to external validation, generalizability, interpretability, and integration into routine radiology workflows continue to limit real-world impact. In particular, radiologist interaction, trust calibration, through strategies such as calibrated confidence scores, standardized reporting of model uncertainty, structured training on AI limitations, and continuous performance feedback, and the limited practical utility of current explainability methods remain under-addressed. Meaningful improvements in lung cancer screening outcomes will therefore depend on deploying AI as a decision-support system that augments clinical expertise, aligns with radiologists’ cognitive workflows, and is validated across diverse populations. With continued progress in federated learning, multi-omics integration, and collaborative infrastructures, AI-driven LDCT screening may evolve into a robust and equitable component of precision lung cancer prevention.
